# Supplementary material for: Anti-LAMP-2 Antibody Seropositivity in Children with Primary Systemic Vasculitis Affecting Medium- and Large-Sized Vessels
Source: Int J Mol Sci. 2024 Mar 28;25(7):3771. doi: 10.3390/ijms25073771 (PMC11011342; doi:10.3390/ijms25073771)
Supplement: Supplementary file 1 [file ijms-25-03771-s001.zip › ijms-2904002-supplementary.pdf]

Supplementary Materials

Supplementary Table S1. Demographic information for participants (n = 18) in the pediatric-onset systemic autoinflammatory disease (SAID) cohort.

**Age**, median (range) in years	8.3 (1.2 - 16.1)
**Female : Male**, n (%)	7 (38.9%) : 11 (61.1%)
**Diagnosis**, n	18
Polygenic (PFAPA, BS, uSAID)	16
Monogenic (TRAPS, FMF)	2
**CRP**, median concentration (range) in mg/L	2.01 (0.1 – 130.7)

CRP: C-reactive protein; normal values <3.0 mg/L, BS: Behcet's Syndrome, FMF: familial Mediterranean fever, PFAPA: periodic fever aphthous stomatitis, pharyngitis and cervical adenitis syndrome, TRAPS: tumor necrosis factor receptor-associated periodic syndrome, uSAID: unclassified systemic autoinflammatory syndrome.
